# Preclinical evaluation of CPL423: a novel potent small-molecule inhibitor of TAM family and FLT3 kinase for cancer therapy

**DOI:** 10.3389/fphar.2026.1768167

**Published:** 2026-03-25

**Authors:** Agata Mikołajczyk, Delfina Popiel, Kinga Jastrzębska, Bartosz Wiernicki, Filip Mituła, Artur Janusz, Jakub Dominowski, Michał Górka, Tomasz Kornatowski, Joanna Hucz-Kalitowska, Małgorzata Teska-Kamińska, Damian Smuga, Monika Delis, Krystian Kamecki, Paweł Maliszewski, Abdellah Yamani, Krzysztof Dubiel, Jerzy Pieczykolan, Maciej Wieczorek

**Affiliations:** 1 Celon Pharma S.A., Preclinical Development Department, Lomianki, Poland; 2 Celon Pharma S.A., Clinical Development Department, Lomianki, Poland; 3 Celon Pharma S.A., Medicinal Chemistry Department, Lomianki, Poland

**Keywords:** TAM kinases, FLT3 Inhibitor, small-molecule inhibitor, cancer therapy, tumor phagocytic capacity, acute myeloid leukemia (AML), CPL423, kinase inhibition

## Abstract

**Introduction:**

The TAM family of receptor tyrosine kinases (TYRO3, AXL, MERTK) promotes tumor survival, metastasis, and immune evasion. Its dysregulation across solid and hematologic cancers is associated with therapy resistance and poor outcomes. FLT3 is a key oncogenic driver in acute myeloid leukemia (AML). We report the preclinical characterization of CPL423, a low-molecular-weight inhibitor of all TAMs and FLT3.

**Method:**

*In vitro* kinase assays quantified potency and kinome selectivity. Antiproliferative effects were measured in FLT3-ITD-driven AML cell lines (MOLM-13, MV4-11). Antitumor efficacy was evaluated in AML xenografts and A375 melanoma (AXL overexpression, BRAF V600E mutation). Phagocytic capacity of antigen presenting cells was addressed using bone marrow derived dendritic cells (BMDC). Physicochemical, ADME/PK, and cardiovascular safety liabilities were profiled.

**Result:**

CPL423 inhibited TAMs and FLT3 with sub-nanomolar IC50s (MERTK 0.47 nM; FLT3 0.94 nM) and high selectivity. It suppressed proliferation in MOLM-13 and MV4-11 (IC50 5.7 and 7.92 nM). In AML xenografts, it achieved up to 98% tumor growth inhibition without observable toxicity; in A375, TGI was 39.4% at 50 mg/kg on day 14 Ex vivo experiments showed that the compound altered the clearance of dying cells by dendritic cells (BMDCs), consistent with TAM-pathway modulation. CPL423 showed high permeability, metabolic stability, and low cardiovascular liability.

**Discussion:**

CPL423 provides direct antitumor activity via dual TAM/FLT3 inhibition and immune-mediated effects on antigen-presenting cells, addressing resistance mechanisms in AML and TAM/AXL-driven solid tumors and supporting further development, including combination regimens.

## 1 Introduction

TYRO3, MERTK, and AXL constitute the TAM family of receptor tyrosine kinases (RTKs), which were identified later due to their initial underestimation as strong oncogenes (Graham et al., 2014). However, growing evidence over the past decade has established TAM receptors as important regulators of tumor biology and immunosuppression, leading to their emergence as promising therapeutic agents.

In their unaltered state, TAM kinases play essential roles in maintaining tissue homeostasis by modulating diverse cellular processes, including survival, migration, cytokine release, phagocytosis, cell proliferation, and stabilization of blood clot formation (Graham et al., 2014; Scott et al., 2001; Schulz et al., 1995; Weinger et al., 2011; Cavet et al., 2008). While these kinases are frequently co-expressed within the same cell types (Rothlin et al., 2015), they exhibit non-redundant, context-dependent functions (Tsou et al., 2014; Dransfield et al., 2016; Zagórska et al., 2014). TAM receptors are broadly expressed across various cell types, including macrophages, dendritic cells, platelets, and epithelial cells (Tirado-Gonzalez et al., 2021; Zhou et al., 2020; Paolino et al., 2014; Giroud et al., 2020). Notably, many TAM-expressing cells also synthesize their ligands (Rothlin et al., 2015), primarily the canonical Gas6 (growth arrest-specific 6) and Protein S (PROS1), as well as noncanonical ligands such as Tubby, Tulp1, and Galectin-3, in a context-dependent manner (Miao et al., 2024; Al Kafri and Hafizi, 2020).

In cancer, aberrant expression of TAM family proteins and their ligands has been documented in both solid tumors and leukemias, particularly in acute myeloid leukemia (AML) (Graham et al., 2006; Shieh et al., 2005; Knubel et al., 2014; Schlegel et al., 2013; Davra et al., 2021; Ben-Batalla et al., 2013; Schm et al., 2016). The altered expression of TAM proteins in tumors typically correlates with adverse clinical outcomes, such as a poor prognosis, increased metastatic potential, and resistance to certain chemotherapeutic agents and targeted therapies (Shieh et al., 2005; Davra et al., 2021; Kimani et al., 2017; Wu et al., 2017; Aehnlich et al., 2021; Morimoto et al., 2020).

Importantly, TAM receptors are not only expressed in tumor cells but are also prominently expressed in immune components of the tumor microenvironment, including tumor-associated macrophages, dendritic cells, and natural killer (NK) cells (Tirado-Gonzalez et al., 2021; Zhou et al., 2020; Giroud et al., 2020; Vaught et al., 2016; Cook et al., 2013; Aguilera et al., 2016). This dual localization underlies their bifunctional role in cancer: within tumor cells, TAM signaling enhances cell survival, proliferation, and resistance to apoptosis; within immune cells, it suppresses innate immune responses, facilitates immune tolerance, and promotes and immunosuppressive microenvironment. Consequently, pharmacologic inhibition of TAM receptors can directly impair tumor growth while also restoring antitumor immunity, thereby enhancing the overall efficacy of anticancer therapies (Tirado-Gonzalez et al., 2021; Davra et al., 2021; Cook et al., 2013; Paolino and Penninger, 2016).

FLT3 (Fms-Related Tyrosine Kinase 3) represents another RTK critically involved in hematologic malignancies. Mutations in the FLT3 gene, particularly internal tandem duplications (FLT3-ITD) and tyrosine kinase domain (TKD) point mutations, are present in approximately 25%–30% of AML cases and are strongly correlated with aggressive disease and poor clinical outcomes (Kennedy and Smith, 2020; Thiede et al., 2002; Schnittger et al., 2002; Fröhling et al., 2002). Beyond AML, FLT3 abnormalities have been implicated in a range of hematopoietic disorders, underscoring its importance as a therapeutic target (Thiede et al., 2002; Schnittger et al., 2002; Fröhling et al., 2002; Tsapogas et al., 2017).

Recent studies indicate functional cross-talk between TAM kinases and FLT3, particularly in AML. TAM receptors, especially AXL, contribute to leukemic phenotypes by activating pro-survival signaling pathways such as PI3K/AKT and MAPK/ERK, and by interacting with FLT3 to enhance its oncogenic activity (Ben-Batalla et al., 2013; Park et al., 2013; Jin et al., 2017). AXL has been identified as a positive regulator of FLT3 activation and is increasingly recognized as a potential mediator of acquired resistance to FLT3-targeted therapies (Huey et al., 2016; Grafone et al., 2012; Myers et al., 2019). The therapeutic relevance of this interaction is highlighted by compounds such as MRX-2843, a dual MERTK/FLT3 inhibitor that demonstrates efficacy against resistance-associated FLT3 mutations and shows promise for AML treatment (Minson et al., 2016). These findings support the rationale for developing agents that co-target both FLT3 and TAM receptors in order to address therapeutic resistance and improve treatment durability.

These findings suggest that simultaneously targeting TAM kinases and FLT3 is a promising therapeutic strategy. FLT3 inhibitors are commonly grouped into type I (DFG-in, active conformation) and type II (DFG-out, inactive conformation) ATP-competitive inhibitors (Hassanein et al., 2016; Zhong et al., 2020; Tong et al., 2020; Wang et al., 2021; Short et al., 2019; Scholl et al., 2020). and differ substantially in binding mode, kinome selectivity, and resistance profiles. Among compounds with documented dual FLT3/TAM activity, gilteritinib (FDA-approved for relapsed/refractory FLT3-mutated AML) is a type I inhibitor with biochemical potency against FLT3 (IC50 = 0.29 nM) and AXL (IC50 = 0.73 nM), and it also inhibits MERTK (IC50 = 1.8 nM) (Mori et al., 2017; Short et al., 2019). Beyond hematologic malignancies, TAM receptors have broad oncogenic functions and have been implicated in tumor invasion and survival across multiple tumor types (Linger et al., 2008; Wang et al., 2013). The therapeutic rationale for co-targeting TAM receptors and FLT3 is further supported by dual MERTK/FLT3 inhibitors such as MRX-2843 (Minson et al., 2016; Wu et al., 2022) and newly reported pyrrolo [2,3-d]pyrimidine-based leads with low-nanomolar inhibition of both MERTK (IC50 = 2.58 nM) and FLT3 (IC50 = 0.86 nM) (Yamani et al., 2026).

Several broader-spectrum TKIs with partial overlap in TAM and FLT3 activity have also been evaluated in AML, including sunitinib (Fiedler et al., 2015) and vandetanib (Macy et al., 2012; Macy et al., 2007), and kinome-wide profiling highlights the promiscuous target engagement typical of first-generation multi-kinase inhibitors (Davis et al., 2011). Beyond these agents, multiple compounds with activity reported against TAM family kinases and/or FLT3 have reached clinical trials, including BMS-777607 [NCT01721148; NCT00792558], TP-0903 [NCT03572634], foretinib [NCT00725712; NCT00725764], merestinib [NCT02920996], ningetinib [NCT02711553], ONO-7475 [NCT03176277, terminated], and MRX-2483 [NCT04872478], while many additional candidates remain at the preclinical stage (Mikolajczyk et al., 2022). Compared with broader-spectrum multi-kinase inhibitors, CPL423 shows markedly reduced activity against PDGFRβ and TRK receptors in the selectivity panel (Figure 2). Despite substantial efforts to develop inhibitors targeting TAM and FLT3 kinases for cancer therapy, our understanding of their effects on the immunosuppressive tumor microenvironment remains limited. To fully harness the therapeutic potential of this dual-targeting approach, further investigation is warranted.

In this study, we present CPL423, a novel small-molecule inhibitor of both TAM family and FLT3 kinases. CPL423 exhibits strong enzymatic inhibition, favorable pharmacokinetic properties, and high activity in FLT3-ITD-driven AML models. In AXL-expressing melanoma, CPL423 also reduced tumor growth, though with more moderate efficacy. In addition to its direct antiproliferative effects, CPL423 decreased the phagocytic activity of bone marrow–derived dendritic cells (BMDCs), suggesting immune-relevant activity *in vitro*; cytokine profiling and immune phenotyping were beyond the scope of this study at this stage of the development. These properties support the further development of CPL423 as a dual-action therapeutic candidate with potential application as monotherapy or in combination regimens, including immune checkpoint inhibitors or chemotherapy, in both hematologic and solid tumors.

## 2 Materials and methods

### 2.1 CPL423 chemical characterization

**FIGURE 1:**
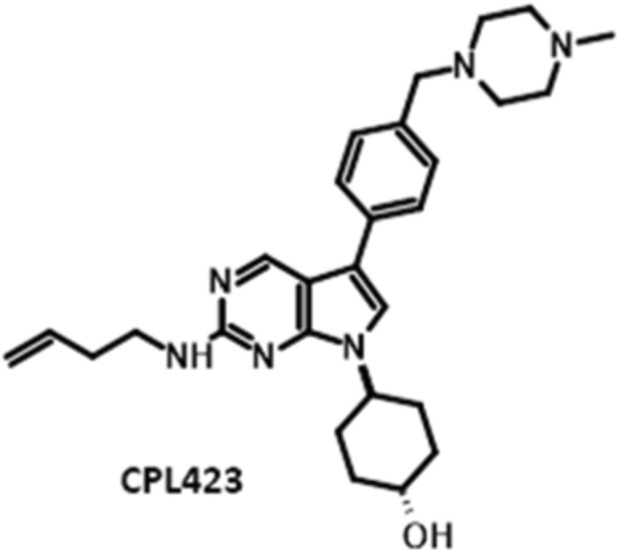
Chemical structure of CPL423 (MW = 474.64).

Compound CPL423, *4-{2-[(but-3-en-1-yl)amino]-5-{4-[(4-methylpiperazin-1-yl)methyl]phenyl}-7H-pyrrolo[2,3-d] pyrimidin-7-yl}cyclohexan-1-ol* (Figure 1), was designed and synthesized by Celon Pharma S.A. (Yamani et al., 2026). For *in vitro* studies, CPL423 was prepared as a 10 mM stock solution in DMSO (Thermo Fisher Scientific) and subsequently diluted in the appropriate assay media. For *in vivo* studies, CPL423 was formulated in a 10% HS15 solution in 0.9% NaCl for oral administration (p.o.) and in acetic buffer (pH 4.5) for intravenous administration (i.v.).

### 2.2 Binding to plasma protein and microsomes

The fraction unbound in plasma (*f*_u,pl_) and in the presence of microsomes (0.5 mg/mL, *f*_u,mic_) was assessed using the rapid equilibrium dialysis (RED) method. The study utilized mouse (CD-1) plasma and microsomes with RED inserts from Gibco, Thermo Fisher Scientific. The experiment was conducted over 4 h at 37 °C, following the protocol outlined in reference (Pankiewicz et al., 2021). Dialysis was performed in PBS (pH 7.4) for *f*_u,pl_ and in 100 mM phosphate buffer (pH 7.4) for *f*_u,mic_, at a 1 µM concentration of the test compounds. Imipramine (IMI) (Merck) and warfarin (WAR) (Thermo Fisher Scientific) were used as reference compounds, representing medium and low *f*_u,pl_, respectively. The concentration of each compound in the samples was quantified using LC-MS.

### 2.3 Metabolic stability

The phase I metabolic stability assay was conducted following a previously published protocol (Gunerka et al., 2020), using human and mouse (CD-1) liver microsomes (Gibco, Thermo Fisher Scientific). Metabolic stability in hepatocytes was evaluated as described previously (Wan and Rehngren, 2006), using mouse (CD-1) hepatocytes (Gibco, Thermo Fisher Scientific) in technical triplicates. Verapamil (VER)(Thermo Fisher Scientific) and warfarin (WAR) were used as reference compounds for high- and low-clearance, respectively. The concentration of each compound in the test samples was determined by LC-MS. Intrinsic clearance (*Cl*_int_) was adjusted for the unbound fraction (*f*_u,mic)_ to derive the unbound intrinsic clearance (*Cl*_int,u_).

To account for the bound fraction, the unbound metabolic stability (*Cl*_int,u_) was calculated by dividing the apparent *Cl*_int_ value by the appropriate correction factor. Only *f*_u,mic_ was used for these calculations, as *f*_u,hep_, and *f*_u,mic_ values are often interchangeable. The *f*_u,mic_ value for VER was sourced from previously published results (Popiel et al., 2024) and assumed to be 0.43.

### 2.4 Caco-2 permeability

Passive permeability (apical to basal, A → B) was assessed in technical triplicates using a 96 well plate format with differentiated Caco-2 cells (CacoReady 96 well, ReadyCell), following previously described methods (Gunerka et al., 2020; Winiwarter et al., 2019). Atenolol (ATE) and propranolol (PRO) were used as reference compounds for low and high permeability, respectively. The concentration of each compound in the respective samples was determined by LC-MS.

### 2.5 hERG channel binding

hERG channel affinity was assessed using the hERG Predictor™ fluorescence polarization kit (Invitrogen, PV5365), following the manufacturer’s instructions.

### 2.6 Analysis of CYP3A4 activity

cDNA synthesis was performed using the SuperScript IV CellsDirect cDNA Synthesis Kit, following the manufacturer’s protocol. After reverse transcription, qPCR was conducted with TaqMan™ Fast Advanced Master Mix and TaqMan probes (TaqMan® Assay probe Hs00430021_m1, catalog number: 4331182, for Human CYP3A4 FAM-MGB and TaqMan® Assay probe Hs01053790_m1, for Human ABCG2 FAM-MGB) for relative quantification of CYP3A4 mRNA and the housekeeping gene β-actin. The detected mRNA levels for CYP3A4 were normalized to β-actin and then further normalized to the 0.1% DMSO control. A parallel plate was used to assess cell viability using the CellTiter-Glo® assay (Promega). A 0.3 µM concentration of CPL423, corresponding to 4.5 µM in plasma, was applied without impacting cell viability.

The effect on CYP3A4 activity was investigated in a differentiated HepaRG cell line (Biopredic International) (Fredlund et al., 2017). Cells were propagated and differentiated according to the manufacturer’s instructions, using validated media supplements provided with the cells (cat. no. ADD710C, ADD720C). Induction of CYP3A4 in cells treated with CPL423 was measured using a luciferase assay and expressed as a relative fold change in enzymatic activity compared to the vehicle control. Transcriptional induction was detected by reverse transcription qPCR and presented as a relative fold change in CYP3A4 mRNA levels, normalized to β-actin mRNA, and then compared to the baseline levels in the vehicle control group (ΔΔCt method).

### 2.7 LC-MS analysis

The content of each analyte in the samples was measured using a UHPLC system (Vanquish Flex, Thermo Fisher Scientific) coupled with a triple quadrupole mass spectrometer (QTRAP 5500+, Sciex) in multiple reaction monitoring (MRM) mode. LC separation was performed on an ACQUITY UPLC BEH C18 column (50 × 2.1 mm, 1.7 µm) with a pre-column (Waters) at 50 °C. The mobile phases were (A) 0.1% (*v*/*v*) formic acid in water and (B) 0.1% (*v*/*v*) acetonitrile: water (9: 10, v/v). Samples were eluted using a mixed-mode gradient: an isocratic flow of 10% B from 0 to 0.5 min, followed by a linear gradient to 95% B at 2.0 min, maintained at 95% B until 3.0 min, and then returning to the initial 10% B at 3.2 min. The column was equilibrated for up to 4.5 min. The flow rate was 0.5 mL/min, and the injection volume was 2 µL. The MRM transition for CPL423 was 475.3 → 375.2 (positive ionization), with a declustering potential (DP) of 200 V, collision energy (CE) of 27 V, and collision cell exit potential (CXP) of 13 V. Imipramine was used as an internal standard. Quantification was achieved using an electrospray ionization interface operating in positive mode for all compounds.

### 2.8 Inhibitor activity

The inhibitory activity of CPL423 against the tested proteins was evaluated using the ADP-Glo assay (Promega) following the manufacturer’s protocol. CPL423 was dissolved in 100% DMSO to prepare a stock solution, and serial dilutions were made with a dilution buffer (20 mM Tris pH 7.5, 10 mM MgCl_2_, 0.1 mM Na_3_VO_4_, 0.01% Triton X-100, 2.5 mM DTT). Kinases (Carna Biosciences) were diluted in buffer (50 mM Tris-HCl pH 7.5, 150 mM NaCl, 10% glycerol, 0.05% Triton X-100, 1 mM DTT). The ATP concentration used in the assay was 30 µM. CSKtide was used as a substrate for MERTK, AXL, TYRO3, TRKA, and TRKC kinases, while SRCtide was used for FLT3, PDGFRβ, and TRKB. Luminescence intensity was measured with a GloMax Discovery (Promega). IC50 values were determined using GraphPad Prism 8 software by fitting individual data points of the curve via non-linear regression (log (inhibitor) vs. normalized response variable slope). Each compound was tested in at least six technical replicates across two separate experiments.

### 2.9 Cell lines

Cell lines were cultured at 37 °C in a humidified 5% CO_2_ incubator and passaged every 2–3 days (Supplementary Table S1). They were regularly tested for *mycoplasma* contamination using the Venor®GeM qEP *Mycoplasma* Detection Kit for qPCR (Minerva Biolabs). The cell seeding density was optimized for each cell line and plate type to ensure logarithmic growth throughout the procedures.

### 2.10 Analysis of cell proliferation and cytotoxicity

Cell viability for A375, MOLM-13, and MV4-11 cell lines was assessed using the MTT (3-(4,5-dimethylthiazol-2-yl)-2,5-diphenyltetrazolium bromide) assay, following the manufacturer’s instructions (Glentham Sciences). Cells were seeded in 96-well white/clear plates (Corning) at 100 µL/well and incubated for 24 h at 37 °C in 5% CO_2_. Drug treatments were performed for 72 h, after which 25 μL of a 5 mg/mL MTT solution was added to each well. The plates were incubated for 4 h at 37 °C. To stop the reaction and dissolve formazan crystals, 100 μL of a solution containing 0.01 M HCl in 10% SDS was added to each well, followed by a 1 h incubation at room temperature. After thorough mixing, absorbance was measured using a MultiScan plate reader (Thermo Fisher Scientific). The experiments were conducted in two biological replicates, and IC50 values were determined using GraphPad 8 software.

Cell viability for the MERTK stable Ba/F3 cell line was evaluated using the CellTiter-Glo® Luminescent Cell Viability Assay (Promega), according to the manufacturer’s instructions. The MERTK stable Ba/F3 cell line was seeded in 384-well white/clear plates (Corning) at 25 µL/well and incubated for 24 h at 37 °C in 5% CO_2_. Drug treatments were administered for 72 h using a 10-point, 3.16-fold (√10; half-log) serial dilution starting at 5 μM, after which CellTiter-Glo® reagent was added to the wells. Plates were shaken at 700 rpm for 2 min and then incubated in the dark at room temperature for 10 min. Luminescence was measured using the GloMax® Discover Microplate Reader (Promega). The experiments were performed in two biological replicates, and IC50 values are reported as mean ± SD, determined using GraphPad Prism 8 software.

For A375 cells, proliferation and cytotoxicity of CPL423 was assessed by fluorescent microscopy. A375 cells were seeded at a density of 2,000 cells/cm^2^ in 96-well plates. On the following day, cells were stained with 1.25 µM Hoechst for 30 min at 37 °C. After staining, cells were washed twice with 100 µL of complete medium and resuspended in medium containing DRAQ7 (1:1000 dilution, Invitrogen, D15106) as a cell death marker and CPL423 at the indicated concentrations. Plates were transferred to a Cytation5 fluorescence microscope (BioTek) for live-cell imaging. Images were acquired every 6 h in both fluorescence and brightfield channels. Total cell numbers were quantified based on the Hoechst signal, while dead cells were identified and quantified using the DRAQ7 signal using the Gen5 software. Each experiment included six technical replicates per condition. Outlier values were excluded based on exploratory data analysis. Final results were visualized using plots generated with custom R scripts.

Phosphatidylserine exposure and plasma membrane permeability were assessed using Annexin V staining. A375 cells were seeded at 2,000 cells/cm^2^ and, on the following day, stimulated with CPL423. Cells were analyzed 72 h post-treatment. Both floating and adherent cells were collected, washed with 1× Annexin V binding buffer (BD Pharmingen, 556454), and stained with Annexin V-FITC (1:100 dilution, Abcam, ab14085) and DRAQ7 (1:1000 dilution). After a 15-min incubation at room temperature in the dark, samples were analyzed using an Attune flow cytometer (Thermo Fisher Scientific). The gating strategy is provided in the Supplementary Figure S3.

### 2.11 Western blot analysis of MERTK pathway proteins

MERTK-stable Ba/F3 cells were seeded at 0.4 × 10*6 cells/mL in 24-well non–tissue-culture-treated plates and incubated for 24 h. CPL423 was added (10× stocks) to yield final concentrations of 1–250 nM (DMSO control) for 1 h. Cells were washed with PBS and lysed in RIPA buffer supplemented with protease and phosphatase inhibitors, EDTA, and clarified by centrifugation. Protein concentration was determined using a BCA assay; equal amounts of protein (20 μg) were resolved by 7.5% SDS–PAGE and transferred to 0.45 μm nitrocellulose membranes. Membranes were blocked (5% milk in TBS-T) and probed with antibodies against phospho-MERTK, total MERTK, phospho-AKT, total AKT, phospho-ERK, total ERK, and β-tubulin, followed by HRP-conjugated secondary antibodies and ECL detection. Experiments were performed in two independent biological replicates.

### 2.12 Isolation and differentiation of mouse bone marrow-derived dendritic cells (BMDCs)

Bone marrow cells were flushed from the femurs and tibias of C57/BL6 mice using RPMI 1640 medium and a syringe with a 26G needle. Following centrifugation (250 x g, 5 min), erythrocytes were lysed by a 5-min incubation in Red Blood Cell Lysing Buffer Hybri-Max (Sigma-Aldrich). The cells were seeded in 10 cm Petri dishes at a density of 5 × 10^6^ cells in 10 mL or BMDC full medium: RPMI 1640 supplemented with 10% FBS, an antibiotic/antimycotic cocktail, 50 μM β-mercaptoethanol (Sigma-Aldrich), and 20 ng/mL mouse granulocyte-macrophage colony-stimulating factor (GM-CSF; Peprotech, 315–03). On the third day of culture, an additional 10 mL BMDC full medium was added. On the sixth day, half of the medium was removed from each plate, centrifuged (250 x g, 5 min), and then the cell pellet was resuspended in 10 mL of BMDC full medium. The cells were cultured for a total of 9 days after seeding.

### 2.13 Efferocytosis analysis

Floating BMDCs were collected and mixed with BMDCs detached by 10 min long incubation with 2 mM EDTA (Thermo Fisher Scientific) at 37 °C. The cells were then washed twice with PBS and resuspended in PBS (1 × 10^7^ cells/ml) with a 1 µM CellTraceViolet (Thermo Fisher Scientific) and incubated for 5 min at 37 °C. Afterwards, cells were washed twice with BMDC full medium and seeded at a density of 5 × 10^4^ cells in 100 µL BMDC full medium in non-TC-treated 96-well plates. BMDCs were supplemented with tested compound were 2 h prior to co-culture while phagocytosis inhibitor Cytochalasin D () was added 1 h prior to co-culture.

Jurkat E6.1 cells were collected, counted, and washed twice with PBS. The cells were then suspended in a 5 µM TAMRA solution (Thermo Fisher Scientific) in PBS at a concentration of 1 × 10^7^ cells/mL and incubated for 5 min at 37 °C. After incubation, the cells were adjusted to a concentration of 1 × 10^6^ cells/mL and incubated overnight at 37 °C. Apoptosis was induced by 4-h incubation with TNF-α (10 ng/mL) and birinapant (1 µM). Finally, apoptotic cells were washed twice with PBS and added to BMDCs at a 1:1 ratio and incubated for 2 h at 37 °C. After incubation, the medium with cells was transferred to a new 96-well plate, and 100 µL of PBS with 2 mM EDTA was added to the cells remaining in the wells. After a 10-min incubation at 37 °C, the contents were mixed and combined with the previously collected medium. The mixture was then centrifuged (450 x g, 3 min), washed with PBS, centrifuged again, and stained with the cell death marker DRAQ7 (1:500) in PBS before analysis using an Attune flow cytometer (Thermo Fisher Scientific). Gating strategy for the experiment is presented in supplementary materials.

### 2.14 Pharmacokinetics

Eight-week-old male BALB/ccmdb mice were obtained from the Centre of Experimental Medicine, Medical University of Bialystok, Poland, where the experiment was conducted. The compound was administered orally (via oral gavage) at a dose of 10 mg/kg body weight and intravenously (via tail vein) at a dose of 1 mg/kg body weight. Blood samples for pharmacokinetic analyses were collected at five different time points (three mice per time point) according to the following schedule: 0.25, 0.5, 2, 4, and 8 h after oral dosing, and 0.08, 0.25, 0.5, 2, and 4 h after intravenous administration. Compound concentrations were determined using LC-MS/MS.

### 2.15 Cell line xenograft models

Eight-week-old female SCID mice (CB-17/Icr-Prkdc^SCID^/Rj) were obtained from Janvier Labs, France. The experiment was conducted at the Hirszfeld Institute of Immunology and Experimental Therapy, Polish Academy of Sciences in Wroclaw. Tumors were established by subcutaneous injection into the left flank with 0.1 mL of tumor cell suspension in PBS (1 × 10^6^ for both A375 and MOLM-13 models), mixed 3:1 with Matrigel (BD Biosciences). Mice were randomized into control and treatment groups (n = 8 mice/group) when tumors reached a size of approximately 100 mm^3^. Tumor volume (measured by Vernier caliper) and animal body weight were recorded twice a week throughout the study. Tumor volume (TV) was calculated using the formula: TV = (L x (W^2^))/2, where L represents the largest dimension and W represents the smallest dimension. Percent Tumor Growth Inhibition (TGI%) was calculated using the formula: TGI [%] = [1− (mean tumor volume of drug-treated group/mean tumor volume of the vehicle-treated control group)] x 100%. Tumor growth inhibition greater than 40% was considered significant (Ubezio, 2019). Animals were treated once daily for 14 days (A375 model) and 11 days (MOLM-13 model) with vehicle placebo or CPL423 by oral gavage at a dose volume of 10 mL/kg. Tolerability was assessed by monitoring body weight loss, clinical signs, and survival. Body weight trajectories are presented in Supplementary Figure S1. Statistical analysis was performed using GraphPad Prism 8 (GraphPad Software). The significance of differences between control and treatment groups was determined using one-way ANOVA, followed by Dunnett’s multiple comparisons test.

### 2.16 Statistical data analysis

GraphPad Prism software (version 8) was used for all statistical analyses of *in vitro* and *in vivo* experiments. Data are presented as means ± SD (for *in vitro* experiments) or means ± SEM (for *in vivo* experiments). IC50 values were calculated based on logarithmic values using nonlinear regression (curve fit) with a four-parameter variable slope equation. Statistical significance between treated groups and the control group was evaluated using one-way or two-way ANOVA, followed by *post hoc* comparisons (Dunnett’s test). *P* values were considered statistically significant as follows: *p < 0.05; **p < 0.01; ***p < 0.001; ****p < 0.0001.

### 2.17 Ethical statements and animal care

All animal experiments were conducted in compliance with the 3Rs (Replacement, Reduction, Refinement) principles and under protocols approved by the respective Local Ethics Committees for Experiments on Animals in Poland:

- Pharmacokinetic study: approval No. 64/2019, University of Warmia and Mazury in Olsztyn, Poland
- Cell Line Xenograft Models: approval No. 070/2020/P1, Hirszfeld Institute of Immunology and Experimental Therapy, Polish Academy of Sciences, Wroclaw, Poland

## 3 Results

### 3.1 CPL423 demonstrates potent inhibitory activity against TAM and FLT3 kinase *in vitro*

To evaluate the basic activity and selectivity of the CPL423, *in vitro* enzymatic assays were conducted against members of the TAM family (MERTK, AXL, and TYRO3), as well as FLT3, TRKA, TRKB, TRKC, and PDGFRβ kinases. In addition to the kinases intentionally targeted by CPL423 (MERTK, AXL, TYRO3, and FLT3), the remaining kinases, PDGFRβ and members of the TRK family, were selected based on earlier broad kinase profiling analyses (data not shown).

**FIGURE 2:**
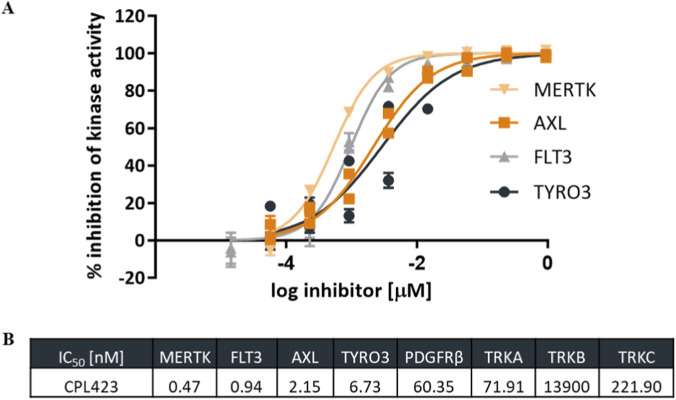
CPL423 inhibits the kinase activity of TAM-family members and FLT3. **(A)** Analysis of the inhibitory activity of CPL423 against MERTK, AXL, TYRO3, and FLT3. The graph displays dose-response curves against 4 kinases obtained using the ADP-Glo assay. The ordinate axis shows percent inhibition, and the abscissa axis represents the logarithm of compound concentration in µM. Error bars indicate SD. **(B)** IC50 values (in nM) for CPL423 against MERTK, AXL, TYRO3, FLT3, PDGFRβ, TRKA, TRKB, and TRKC kinases.

These results confirmed CPL423’s potent inhibitory activity, with subnanomolar IC50 values for MERTK (0.47 nM) and FLT3 (0.94 nM). Additionally, CPL423 exhibited low nanomolar IC50s for two other members of the TAM family, AXL and TYRO3 (2.15 and 6.73 nM, respectively) (Figure 2).

To assess off-target activity, CPL423 was also tested against PDGFRβ and members of the neurotrophin receptor family (TRKA, TRKB, and TRKC), which are frequently inhibited by TAM-targeting compounds. CPL423 demonstrated markedly reduced activity against these kinases, with IC50 values of 60.35 nM for PDGFRβ, 71.91 nM for TrkA, 221.90 nM for TrkC, and 13900 nM for TrkB (Figure 2).

Within the tested kinase panel, CPL423 showed a favorable selectivity profile, with potent activity toward the TAM receptors and FLT3 and notably lower inhibition of unrelated kinases.

### 3.2 CPL423 demonstrates potent inhibition of TAM and FLT3 kinase activity in cell lines

To evaluate the *in vitro* efficacy of CPL423, its cytotoxic activity was tested across selected human cancer cell lines characterized by varying expression of TAM receptors (AXL, MERTK, TYRO3) and FLT3 (Supplementary Table S1). These included the MOLM-13 and MV4-11, which both harbor FLT3-ITD mutations, and the A375 melanoma line, which express high levels of AXL but lacks TAM-activating mutations. In addition, Ba/F3 cells expressing recombinant human MERTK were used as a defined system to confirm MERTK-specific activity.

**FIGURE 3:**
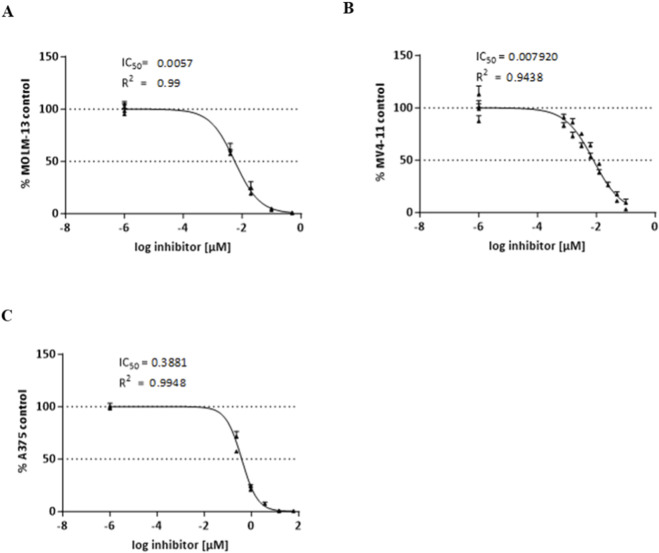
Effect of CPL423 on the viability of cancer cell lines (MOLM-13, MV4-11, A375). Graphs **(A–C)** show dose-response curves from MTT assays after 72 h of treatment with CPL423 for MOLM-13 (AML) **(A)**, MV4-11 (AML) **(B)**, and A375 (melanoma) **(C)** cell lines. IC50 values are expressed in µM, with error bars representing SD of at least three independent experiments.

**FIGURE 4:**
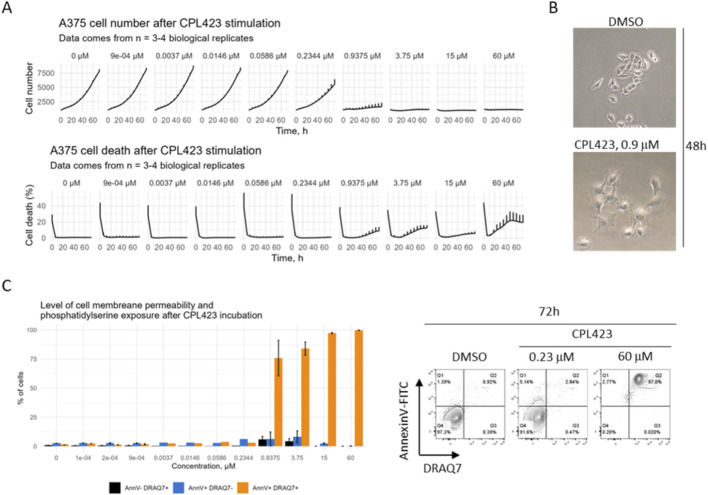
Antiproliferative and cytotoxic properties of CPL423. **(A)** A375 cells were stimulated with various concentrations of CPL423 and analyzed in terms of the cell number and cell death in the function of time. Data presented as mean±SD from n = 3-4 biological replicates. **(B)** Cell morphology 48 h after CPL423 stimulation. Medium dosage of the compound results in senescent – like morphology of the cells. **(C)** The analysis of phosphatidylserine exposure and cell membrane permeability. A375 cells were stimulated with CPL423 compound for 72 h and stained with Annexin V and DRAQ7. Data comes from n = 3 biological replicates.

In AML models, CPL423 exhibited potent, dose-dependent cytotoxic activity. The IC50 was 5.7 nM in MOLM-13 cells (Figure 3A) and 7.92 nM in MV4-11 (Figure 3B). In the A375 melanoma cell line, CPL423 reduced cell viability with an IC50 of 388.1 nM (Figure 3C). Next, we performed the analysis of A375 cells proliferation and viability in the function of time after incubation with various concentrations of CPL423. Our results showed, that stimulating the cells with concentrations of the compound ∼1 µM or higher resulted in cell growth retardation and increased levels of cell death (Figure 4A). Additionally, we observed morphological changes in the cells, specifically increased size and irregular shape suggesting senescence (Figure 4B). Further analysis showed that the affected cells had very low levels of phosphatidylserine exposure suggesting a mode of cell death that is not purely apoptotic (Figure 4C). These data suggest, that the main mode of action in A375 cell line is cell growth inhibition.

**FIGURE 5:**
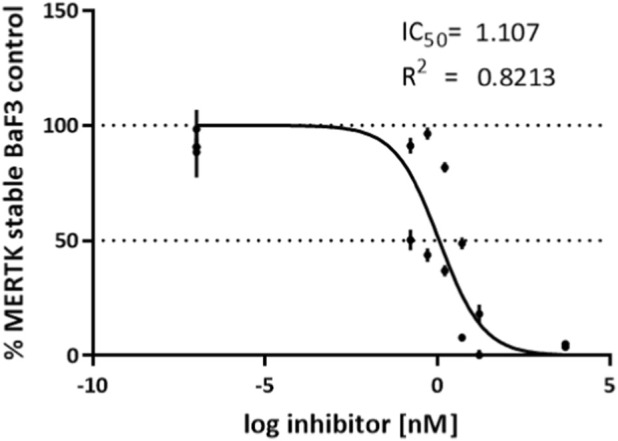
CPL423 potently reduces viability of MERTK-expressing Ba/F3 Cells. Dose–response curve showing the effect of CPL423 on the survival of Ba/F3 cells stably expressing MERTK. IC50 values were determined using the CellTiter-Glo assay after 3 days of compound incubation. Results are expressed in µM. Error bars represent SD.

Treatment of Ba/F3 MERTK-expressing cells with CPL423 resulted in significant inhibition of cell viability, with an IC50 of 1.11 nM (Figure 5), confirming the compound’s high potency. This result demonstrates that CPL423 is a highly effective and impairs the survival of MERTK-driven cells at low nanomolar concentrations.

Target engagement and pathway inhibition at protein level were assessed by Western blotting in MERTK-stable Ba/F3 cells. CPL423 reduced MERTK phosphorylation (pMERTK) in a dose-dependent manner across 1–250 nM, while total MERTK levels remained largely unchanged. Consistent with TAM downstream signaling blockade, phosphorylation of AKT and ERK was concomitantly reduced, whereas total AKT and ERK levels were not markedly affected; β-tubulin served as a loading control. Representative immunoblot is shown in Supplementary Figure S2.

### 3.3 CPL423 exhibits favorable *in vitro* ADME properties

Following the promising efficacy of CPL423 in *in vitro* models, a comprehensive assessment of its ADME properties was performed to evaluate its potential physiological behavior and suitability as a therapeutic agent.

**FIGURE 6:**
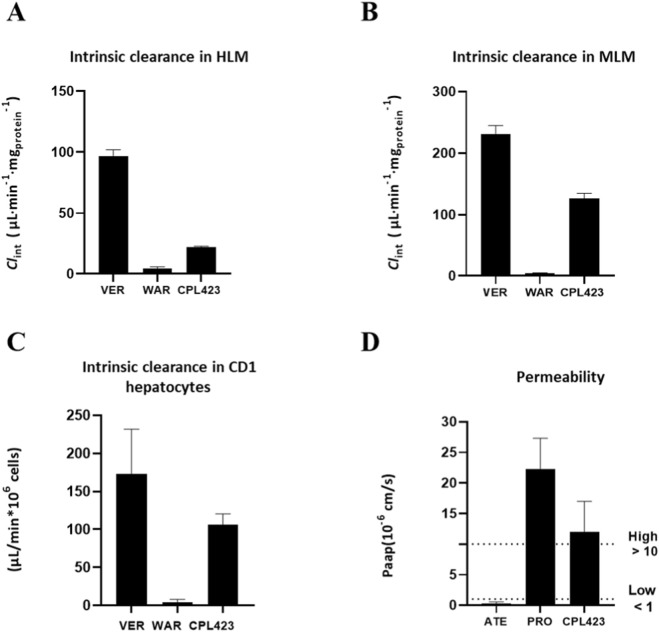
Metabolic stability and permeability of CPL423. ADME parameters were assessed to evaluate the *in vitro* bioavailability of CPL423. Panels include: Intrinsic clearance in **(A)** human liver microsomes (HLM), **(B)** mouse liver microsomes (MLM), **(C)** mouse CD1 hepatocytes, and **(D)** permeability through the Caco-2 cell monolayer. Bars represent mean values for CPL423 and control compounds. Error bars indicate SD. Control compounds are verapamil (VER), warfarin (WAR), atenolol (ATE), and propranolol (PRO).

Metabolic stability was first assessed using standard microsomal stability assays in human (HLM) and mouse (MLM) liver microsomes. This widely accepted model allows evaluation of xenobiotic metabolism in a biologically relevant system. The intrinsic clearance (*Cl*_int_) of CPL423 was calculated to estimate metabolic stability, with verapamil (VER) and warfarin (WAR) serving as reference compounds representing low and high metabolic stability, respectively. Microsomal clearance values for CPL423 and the reference compounds are summarized in Figures 6A–C and Supplementary Table S2. In these assays, CPL423 showed Cl_int_ values of 21.9 μL/min*mg in HLM and 126.5 μL/min*mg in MLM, indicating favorable metabolic stability. Additionally, passive permeability of CPL423 was evaluated using the transwell Caco-2 *in vitro* model, focusing on apical-to-basal (A → B) transport. This model assesses the compound’s ability to permeate cellular membranes, a key determinant of oral bioavailability. Apparent permeability coefficients (P_app_ AB) for CPL423 and control compounds are presented in Figure 6D and Supplementary Table S3. The P_app_ AB value for CPL423 was determined to be 9.51 × 10^−6^ cm/s. These results indicate favorable permeability characteristics, suggesting potential for effective oral absorption.

Overall, the ADME profile of CPL423 demonstrates satisfactory metabolic stability and passive permeability, supporting its further development as a viable therapeutic candidate.

### 3.4 CPL423 exhibits low cardiovascular and hepatotoxicity risk *in vitro*

**FIGURE 7:**
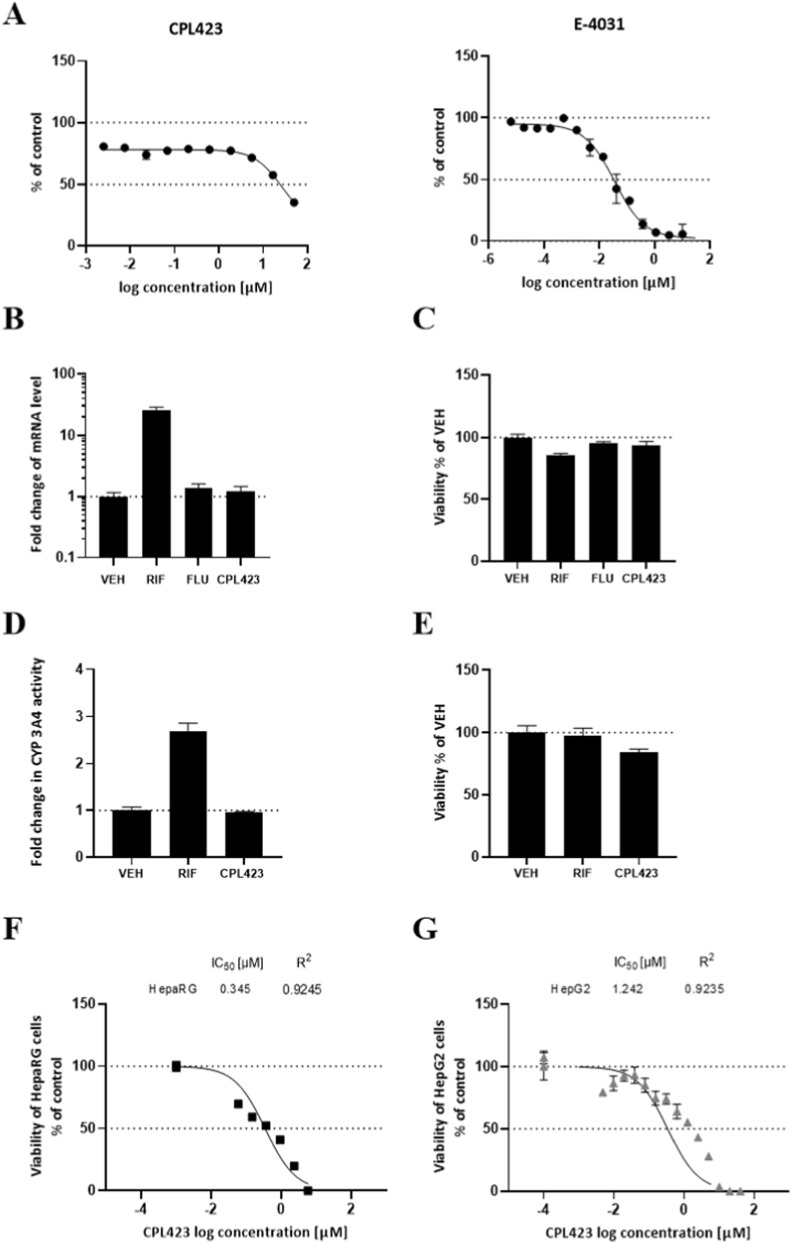
Evaluation of CPL423 cardiac and hepatic toxicity. **(A)** The hERG channel affinity study indicates a low binding potential of CPL423 compared to the high-affinity control E-4031. **(B)** CPL423 does not induce the CYP3A4 isoform of cytochrome P450 in differentiated HepaRG cells at a biologically relevant concentration of 0.3 μM, as assessed by transcription. **(C)** The viability test confirms that CPL423 does not cause toxic effects at this concentration. **(D)** CPL423 also does not affect CYP3A4 enzymatic activity. **(E)** The viability test further supports the absence of toxic effects at the tested concentration. **(F)** and **(G)** High concentrations of CPL423 result in toxic effects on differentiated HepaRG cells and HepG2 cell cultures, indicating potential dose-dependent hepatotoxicity. Bars represent mean values with SD. Control compounds include vehicle (VEH), rifampin (RIF), and flumazenil (FLU).

Prior to conducting *in vivo* studies, the compound’s potential toxicity was evaluated *in vitro*, assessing possible cardiovascular and hepatic effects. To assess cardiotoxicity, the compound’s affinity for the hERG potassium channel was examined in a dose-dependent assay. Compared to the reference compound E-4031, a high-affinity hERG inhibitor (IC50 = 35.58 nM), CPL423 exhibited a very weak affinity for the hERG channel (IC50 = 27 µM) (Figure 7A). These data suggest a minimal risk of myocardial toxicity associated with CPL423. Hepatotoxicity was first assessed using the HepaRG cell line in a *CYP3A4* gene induction assay. Since CYP3A4 is a key enzyme involved in xenobiotic metabolism, its induction may indicate potential liver toxicity. CPL423, tested at a biologically relevant concentration of 0.3 µM, did not induce CYP3A4 mRNA expression compared to the positive control Rifampin or the non-inducer Flumazenil (Figure 7B). Additionally, no changes in CYP3A4 enzymatic activity were observed (Figure 7D), and cell viability was unaffected at this concentration (Figures 7C,E).

To further evaluate hepatotoxicity, a luciferase-based ATP assay was conducted in HepG2 and differentiated HepaRG cells. CPL423 showed IC50 values of 0.345 µM for HepaRG cells and 1.242 µM for HepG2 cells (Figures 7F,G), indicating a possible low cytotoxicity at therapeutically relevant concentrations.

### 3.5 CPL423 reduces the phagocytic capacity of bone marrow-derived mouse dendritic cells (BMDCs)

**FIGURE 8:**
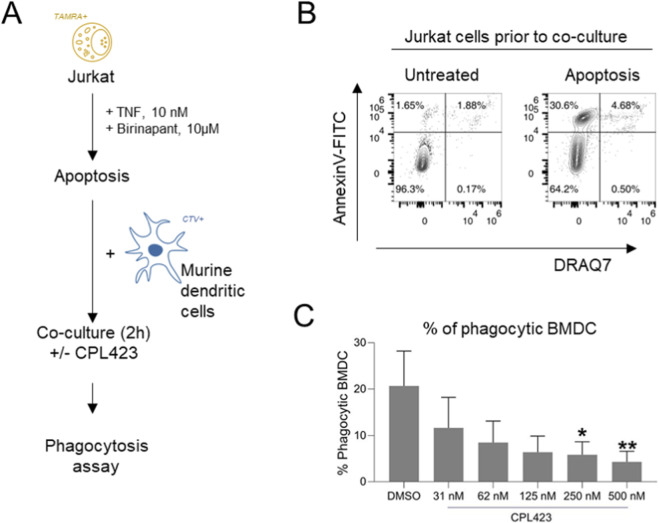
CPL423 attenuates apoptotic cell clearance by BMDCs. **(A)** Schematic overview of the experimental design. **(B)** Phosphatidylserine exposure and membrane permeabilization of Jurkat E6.1 cells following stimulation with TNF-α and birinapant, representative of n = 10 experiments. **(C)** Phagocytic activity of BMDCs after 2-h of co-culture with apoptotic Jurkat cells in the presence of increasing concentrations of CPL423.

Based on the CPL423s profile the potential inhibitory effect of the compound on the efferocytic properties of antigen-presenting cells was examined. Co-culture of BMDCs and apoptotic Jurkat cells (Figures 8A,B) enriched with CPL423 significantly impaired the number of phagocytic BMDCs. The observed effect was dose-dependent. The reduction in phagocytic capacity was statistically significant starting at 250 nM CPL423 (Figure 8C). The results clearly demonstrate that CPL423 blocks efferocytosis of early apoptotic cells (i.e., the engulfment and clearance of apoptotic cells by phagocytes) by antigen presenting cells in a murine model.

### 3.6 CPL423 exhibits a favorable pharmacokinetic profile

**FIGURE 9:**
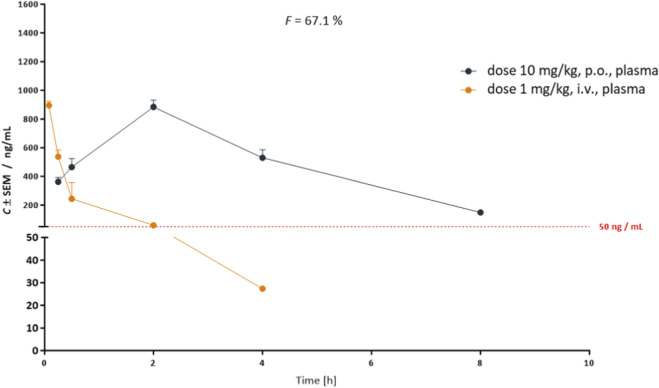
Pharmacokinetic profile of CPL423 in mice. Plasma concentration-time curves of CPL423 following intravenous (i.v.) and oral (p.o.) administration in BALB/c mice (n = 5 per time point). The y-axis indicates compound concentration (ng/mL), and the x-axis represents time (hours, h) post-administration. Error bars indicate SEM. F denotes oral bioavailability.

The pharmacokinetic (PK) profile of CPL423 was evaluated in BALB/c mice (n = 5) following oral (10 mg/kg) and intravenous (1 mg/kg) administration (Figure 9; Supplementary Table S4). CPL423 showed substantial oral exposure (3935.6 ng*h/mL) with a high plasma clearance rate of 2.25 L/h/kg, resulting in a plasma half-life of approximately 2 h. The compound demonstrated an oral bioavailability of 67.1% (Figure 9). Full data is available in Supplementary Table S4.

### 3.7 CPL423 demonstrates antitumor activity in xenograft models

The antitumor activity of CPL423 was evaluated in two subcutaneous human tumor xenograft models characterized by alterations or expression within the TAM family or FLT3: MOLM-13, a human acute myeloid leukemia cell line with a heterozygous FLT3-ITD mutation, and A375, a human melanoma cell line with high AXL expression. These distinct TAM/FLT3-relates tumor models were selected to explore tumor histology that may respond to CPL423, with the aim of guiding future patient stratification in clinical settings.

**FIGURE 10:**
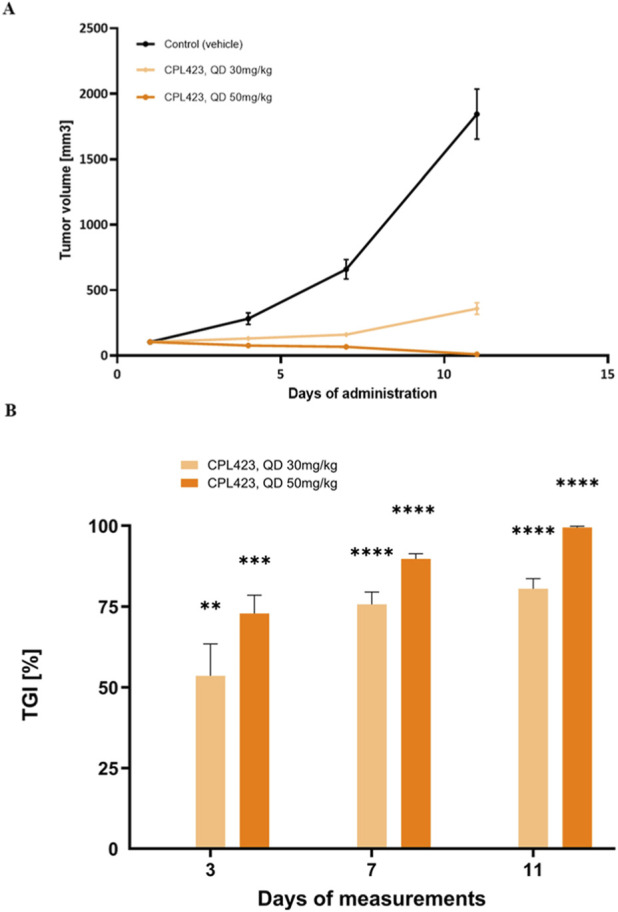
Efficacy of CPL423 in the MOLM-13 acute myeloid leukemia xenograft model. **(A)** Tumor growth kinetics in MOLM-13 xenografts following oral administration of CPL423 once daily at 30 mg/kg and 50 mg/kg body weight. The graph shows tumor volumes (mm^3^) recorded on consecutive measurement days. The y-axis represents tumor volume; error bars indicate SEM. **(B)** Tumor growth inhibition (TGI) over time in response to CPL423 treatment. The graph displays percentage TGI on each measurement day, with data shown through day 11, when animals in the vehicle control group were euthanized. p < 0.05, **p < 0.01, ***p < 0.001, ****p < 0.0001 vs. vehicle control.

**FIGURE 11:**
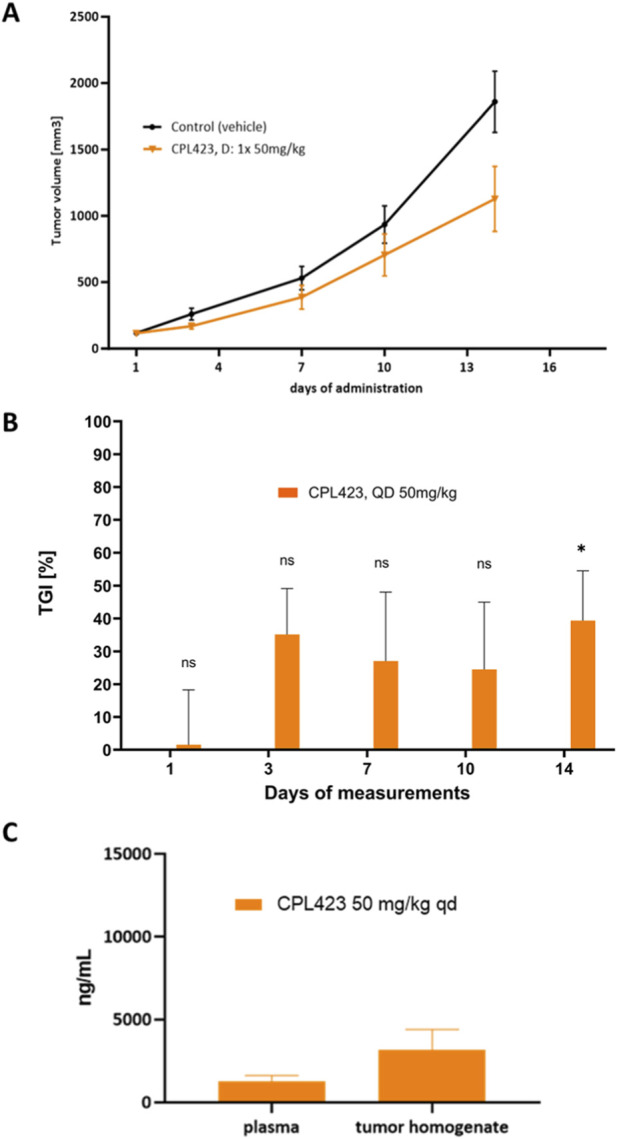
Efficacy of CPL423 in the A375 melanoma xenograft model. **(A)** Tumor growth kinetics in A375 xenografts. Results are presented as means (n = 8); error bars represent SEM. **(B)** Tumor growth inhibition (TGI) in A375 xenografts in response to CPL423 treatment. The graph displays percentage TGI relative to the control group on consecutive measurement days (n = 8), *p < 0.05 vs. control. **(C)** CPL423 concentrations in plasma (n = 8) and tumor homogenates (n = 3), measured 2 h after the final dose. Error bars represent SD.

CPL423 or vehicle was administered orally, once daily (QD), at doses ranging from 30 to 50 mg/kg. In the MOLM-13 xenograft model, CPL423 strongly inhibited tumor growth in a dose-dependent manner. Both treatment groups (30 and 50 mg/kg) showed statistically significant tumor growth inhibition (TGI) compared to the vehicle group (Figure 10A). On day 11 of treatment (end of the study), the TGI for the 30 mg/kg group reached 98% (Figure 10B). A markedly weaker effect of CPL423 was observed in the A375 model with AXL expression (Figure 11A). At the 50 mg/kg dose, a statistically significant reduction in tumor size was achieved, with a tumor growth inhibition (TGI) of 39.4% observed on day 14, which marked the end of the study (Figure 11B). In the A375 model, CPL423 concentrations were measured in plasma and tumor homogenates 2 h after the final oral dose of 50 mg/kg (QD) (Figure 11C). The compound was detectable in both compartments, with higher concentrations observed in tumor tissue compared (3185.5 ng/mL) to plasma (1265.33 ng/mL), indicating accumulation of CPL423 at the tumor site (Figure 11C).

In both xenograft studies, major organs (heart, liver, kidneys, lungs, and spleen) were collected at study termination for H&E histopathological assessment, and blood was collected for hematology and clinical chemistry analyses. No significant body weight loss (Supplementary Figure S1) or general toxicity (defined as morbidity or non-specific clinical signs) was observed in either model, and hematological parameters did not show substantial alterations (data not shown).

## 4 Discussion

TAM receptors have emerged as promising targets for cancer treatment, with several low molecular-weight (LMW) inhibitors already in use or in development. Many of these compounds also inhibit FLT3 kinase, an oncogene implicated in hematologic malignancies and some solid tumors (Minson et al., 2016; Mori et al., 2017). Targeting both TAM receptors and FLT3 opens new therapeutic avenues, particularly for resistant cancers in which these pathways are co-activated or compensate for each others’ inhibition (Park et al., 2015; Dumas et al., 2019; Seale et al., 2025).

In this study, we introduce CPL423, a novel LMW inhibitor that efficiently targets all three TAM family members (TYRO3, MERTK, AXL) as well as FLT3. CPL423 demonstrated high potency *in vitro*, with low- and sub-nanomolar IC50 values for MERTK (0.47 nM), AXL (2.15 nM), and TYRO3 (6.73 nM), as well as FLT3 (0.94 nM). These data are consistent with the established structural similarities among TAM and FLT kinases (Graham et al., 1995; Liu et al., 2017; Geng et al., 2017; Nice et al., 1997), which present challenges for developing isoform-selective inhibitors (Davies et al., 2000; Bain et al., 2007; Arter et al., 2022). As observed with several inhibitors in this class, CPL423 displayed the highest affinity for MERTK and AXL, with comparatively lower potency against TYRO3 (Rios-Doria et al., 2020) – a trend commonly attributed to subtle differences in the kinase domain topography (Liu et al., 2017).

CPL423 was designed on the 7H-pyrrolo [2,3-d]pyrimidine core - a compact, synthetically tractable hinge-binding motif that enables systematic tuning of potency and selectivity across closely related kinase active sites. Building on the therapeutic rationale for co-targeting FLT3 and TAM kinases to address resistance mechanisms in AML (Ben-Batalla et al., 2013; Park et al., 2013; Jin et al., 2017; Huey et al., 2016; Grafone et al., 2012; Myers et al., 2019; Ezelarab et al., 2023; Ezelarab et al., 2024) and on dual-target precedents in this space (Minson et al., 2016; Wu et al., 2022), we used this scaffold to retain a conserved hinge-binding element while projecting substituents into solvent-exposed regions to balance target coverage and drug-like properties. In our SAR program, the core was maintained and three regions were diversified: (i) the C5 aryl/benzyl vector to modulate TAM selectivity, (ii) polar solvent-exposed substituents (e.g., piperazinyl and alcohol-bearing side chains), to improve physicochemical properties and exposure, and (iii) the C2 amine substituent to tune FLT3 potency and cellular activity. These modifications were guided by structure-based bioisosterizm and molecular hybridization, complemented by machine learning (ML) algorithms assisted IC50 prioritization, and were designed to differentiate CPL423 from existing FLT3/AXL inhibitors such as gilteritinib (Mori et al., 2017) and broader multi-kinase agents such as cabozantinib (Grüllich and Martens, 2018). Collectively, this scaffold-driven optimization addresses the problem investigated here by combining pan-TAM inhibition with FLT3 blockade in a single chemotype, aiming to suppress FLT3-driven oncogenic signaling while limiting TAM-mediated bypass signaling that contributes to treatment escape (Ben-Batalla et al., 2013; Park et al., 2013; Jin et al., 2017; Huey et al., 2016; Grafone et al., 2012; Myers et al., 2019; Minson et al., 2016).

Highly optimized structure of CPL423 resulted in strong selectivity within the panel of kinases tested, showing minimal off-target activity at concentrations above 60 nM, with only modest inhibition of PDGFRβ and TRKA. This selectivity profile suggests a potentially favorable safety margin. Broader kinome profiling would be valuable for further confirmation of the selectivity of CPL423 and for excluding potential off-target liabilities prior to clinical translation.

In cell-based assays, CPL423 effectively inhibited proliferation in MOLM -13 (IC50 = 5.7 nM) and MV4-11 (IC50 = 7.92 nM) cells, two acute myeloid leukemia cell lines that harbor internal tandem duplications in FLT3 (FLT3-ITD) and are highly dependent on FLT3 signaling (Seipel et al., 2018). These findings are consistent with the well-established role of FLT3-ITD as one of the major oncogenic drivers in AML (Thiede et al., 2002). FLT3-ITD mutations, present in approximately 25% of AML cases, are associated with poor clinical outcomes and represent a key therapeutic target in this disease context (Gilliland and Griffin, 2002). MOLM-13 and MV4-11 represent genetically and functionally relevant preclinical models for evaluating FLT3-targeted agents. CPL423’s potent *in vitro* activity translated into robust antitumor efficacy *in vivo*, as demonstrated in the MOLM-13 xenograft model. Oral administration of CPL423 resulted in dose-dependent tumor growth inhibition, with up to 98% TGI observed at 30 mg/kg. The compound was well tolerated, with no evidence of systemic toxicity, supporting its suitability for further development for FLT3-mutant AML.

In contrast, the A375 melanoma cell line, characterized by an activating BRAF V600E mutation, high levels of AXL protein expression, and no known genetic alterations in AXL, displayed markedly lower sensitivity to CPL423 *in vitro* (IC50 = 388.1 nM). *In vivo*, moderate tumor growth inhibition (39.4% TGI) was observed at the dose 50 mg/kg. These results suggest that although AXL is overexpressed, the primary oncogenic driver in A375 is BRAF, and thus tumors may not be fully dependent on AXL signaling for survival or progression. Notably, AXL has been implicated in both intrinsic and acquired resistance to BRAF inhibitors in melanoma by promoting phenotypic plasticity and EMT. Nyakas et al. have demonstrated that combining an AXL inhibitor with BRAF-targeted therapy significantly improved efficacy in AXL high with BRAF V600E models (Nyakas et al., 2022). This indicates that co-targeting AXL and BRAF may enhance therapeutic outcomes. Because both xenograft experiments were conducted in SCID mice lacking functional T and B cells, immune-mediated and microenvironmental contributions of TAM inhibition may be underestimated in this setting. To bridge this gap, follow-up studies in immune-reconstituted (humanized) models and combination studies with BRAF/MEK inhibition in AXL-high BRAF V600E melanoma are warranted.

CPL423 was well tolerated *in vivo*, showing no toxicity even at the highest tested dose (50 mg/kg) and CPL423 levels were substantially higher in the tumor homogenates than in plasma. This suggests that CPL423, while having limited therapeutic activity alone, has a potential to be used in combination strategies. Additionally, CPL423 strongly reduced the viability of Ba/F3-MERTK cells in a dose-dependent manner, with an IC50 of 1.11 nM. These findings confirm on-target activity in a MERTK-driven cellular system and further supports the compound’s potency against this TAM family member. The use of Ba/F3 cells, which depend exclusively on the introduced kinase for survival, provides a stringent model for evaluating target engagement and further strengthens the rationale for CPL423 as a TAM-directed therapeutic agent.

Importantly, targeting TAM receptors such as MERTK and AXL has gained increased recognition not only for its direct inhibition of pro-oncogenic signaling but also for its pivotal role in shaping the immunosuppressive tumor microenvironment. In addition to direct antiproliferative effects, CPL423 inhibits dead cell clearance by dendritic cells, a process observed in tumor microenvironment and contributing to tolerance induction. This observation aligns with evidence that TAM receptor inhibition reprograms myeloid cells toward pro-inflammatory phenotypes, reducing immunosuppressive cytokines and tumor angiogenesis (Aehnlich et al., 2021; Chantrain et al., 2008). By modulating the abundance and activity of tumor-associated dendritic cells, CPL423 may promote a shift toward a more immunogenic tumor milieu, thereby enhancing responses to existing immune checkpoint inhibitors, such as PD-1/PD-L1-directed therapies (Msaouel et al., 2021; Yang et al., 2025). This dual mechanism - combining direct antitumor activity with immune modulation - reflects an emerging paradigm in the development of next-generation cancer therapeutics. Cytokine/chemokine profiling and immune-cell phenotyping were not performed in this study; therefore, mechanistic conclusions regarding *in vivo* immune reprogramming remain to be established.

In addition to its potent kinase inhibition profile, CPL423 demonstrated favorable drug-like properties that support its development as an orally bioavailable therapeutic candidate. The compound showed good metabolic stability in both human and mouse liver microsomes, with intrinsic clearance values within a range generally considered acceptable for small molecules. Moreover, CPL423 exhibited high passive permeability in the Caco-2 transwell model (P_app_ A→B = 9.51 × 10^−6^ cm/s), which is indicative of efficient gastrointestinal absorption and suggests good oral bioavailability. These findings are consistent with the observed pharmacokinetic profile in mice, where oral administration resulted in substantial plasma exposure and a bioavailability of over 67%.

From a safety perspective, CPL423 displayed a low risk of cardiotoxicity, with minimal binding affinity to the hERG potassium channel (IC50 = 27 µM), a common off-target concern for kinase inhibitors. Regarding hepatic safety, CPL423 did not induce CYP3A4 mRNA expression or enzymatic activity in HepaRG cells at 0.3 µM. However, reduced cellular ATP levels were observed in HepaRG and HepG2 cells at higher concentrations (IC50 = 0.345 µM and 1.242 µM, respectively), indicating a potential for dose-dependent hepatotoxicity. Importantly, HepaRG and HepG2 models, while widely used, do not fully replicate *in vivo* hepatic complexity, and further toxicological evaluations in animal models will be required to assess long-term hepatic safety.

However, at higher concentrations, CPL423 reduced ATP levels in both HepaRG and HepG2 cells (IC50 = 0.345 µM and 1.242 µM, respectively), suggesting possible dose-dependent hepatocellular stress. Although this effect was observed at concentrations above those used in other functional assays, it warrants attention in subsequent *in vivo* safety studies. It is also important to acknowledge that *in vitro* hepatocyte models - while widely utilized - do not fully replicate the metabolic complexity and adaptive responses of the human liver *in vivo*.

Taken together, these findings indicate that CPL423 has a manageable *in vitro* safety profile, with minimal cardiotoxic risk and low hepatotoxic potential within the tested range. Nonetheless, further *in vivo* toxicology studies will be essential to define its safety margins and therapeutic window.

Further investigation using translational models, such as patient-derived xenograft (PDX) and humanized mouse systems, and combination strategies will be essential to define its optimal therapeutic window and elucidate its ability to overcome resistance mechanisms that compromize the durability of current targeted therapies. While the present work establishes CPL423 as a potent and selective dual TAM/FLT3 inhibitor with *in vivo* efficacy in FLT3-ITD AML, several factors currently limit immediate clinical translation. First, the *in vivo* efficacy evidence is restricted to immunodeficient xenografts, so the contribution of immune-mediated mechanisms cannot be quantified *in vivo*. Second, cytokine/chemokine profiling and deeper immune phenotyping were not performed, and broader kinome-wide selectivity profiling would further de-risk off-target liabilities. Third, clinical translation will require an integrated PK/PD package (including exposure–target engagement relationships in tumor and relevant immune compartments) and formal safety studies (GLP toxicology and safety pharmacology) to define the therapeutic window. Future research should therefore prioritize (i) evaluation in immune-reconstituted and patient-derived models with biomarker-driven stratification (e.g., FLT3-mutant AML and TAM-high solid tumors), (ii) mechanism-focused immune readouts (cytokine panels and flow-cytometry-based immune profiling), and (iii) rational combination studies (e.g., with standard AML regimens or, in AXL-high BRAF V600E melanoma, with BRAF/MEK inhibition and/or immune checkpoint blockade) to determine where dual TAM/FLT3 inhibition offers the greatest clinical value. Overall, further research is needed to optimize both efficacy and safety, by refining exposure–response relationships, confirming selectivity in broader profiling, and defining a robust therapeutic window for CPL423.

Still, our data demonstrate CPL423 is a potent dual inhibitor of TAM kinases and FLT3, showing strong antitumor activity, favorable selectivity, oral bioavailability, and a manageable *in vitro* safety profile. Its ability to also alter efferocytosis in immune cells suggest the dual activity mechanism and supports its potential use in both hematologic malignancies and solid tumors (e.g., AML, NSCLC). Overall, CPL423 represents a promising candidate for advancing TAM- and FLT3-targeted precision therapies.

## Data Availability

The raw data supporting the conclusions of this article will be made available by the authors, without undue reservation.
