# Supplementary material for: Preclinical evaluation of CPL423: a novel potent small-molecule inhibitor of TAM family and FLT3 kinase for cancer therapy
Source: Front Pharmacol. 2026 Mar 25;17:1768167. doi: 10.3389/fphar.2026.1768167 (PMC13058478; doi:10.3389/fphar.2026.1768167)

**Supplementary Figure S1. Body weight during xenograft studies (CPL423).**

A) A375 xenograft (QD oral dosing; CPL423 50 mg/kg vs vehicle).





B) MOLM-13 xenograft (QD oral dosing; CPL423 30 and 50 mg/kg vs vehicle).





*Note: This supplementary figure includes body-weight data only for CPL423; raw study reports contained additional comparator compounds and are therefore not provided in full.*

**Supplementary Figure S2. CPL423 reduces MERTK phosphorylation (pMERTK) in a dose-dependent manner.** Total MERTK levels remain unchanged. Phosphorylation of AKT and ERK are reduced, whereas total AKT and ERK levels are not markedly affected;


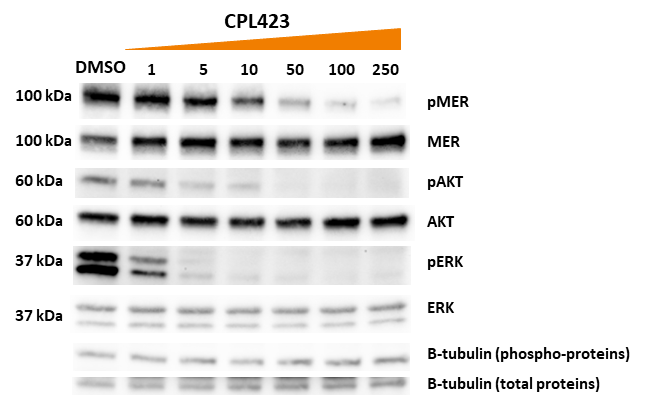


**Supplementary Figure S3. Gating strategy for AnnexinV and DRAQ7 measurement in A375 cells.**


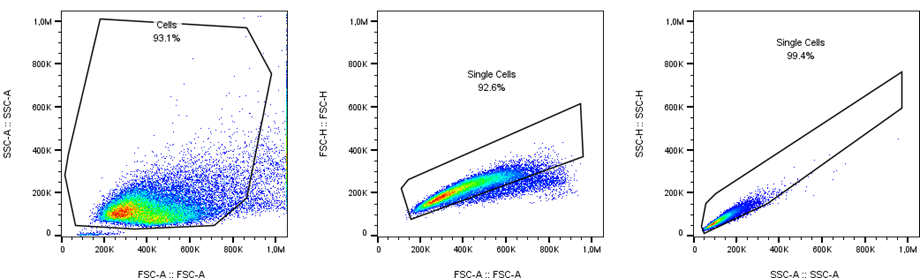

Supplement: Supplementary file 1 [file Supplementaryfile1.docx]
