# Supplementary material for: Preclinical evaluation of CPL423: a novel potent small-molecule inhibitor of TAM family and FLT3 kinase for cancer therapy
Source: Front Pharmacol. 2026 Mar 25;17:1768167. doi: 10.3389/fphar.2026.1768167 (PMC13058478; doi:10.3389/fphar.2026.1768167)
Supplement: Supplementary file 2 [file Table1.docx]

Supplementary Material

**Table S1. List of cell lines**

| **Cell line** | **Cat no., supplier** | **Tissue, disease** | **TAM family and FLT3 expression** |
| --- | --- | --- | --- |
| **Human tumor cell lines** | | | |
| A375 | CRL-1219, ATCC | Skin, melanoma | AXL, TYRO3 |
| MOLM-13 | ACC 554, DSMZ | Blood, AML | FLT3 (ITD) |
| MV4-11 | ACC 102, DSMZ | Blood, AML | FLT3 (ITD) |
| Jurkat E6.1 | TIB-152, ATCC | Blood, T-CLL | MERTK |
| **Mouse MERTK-dependent cell line** | | | |
| MERTK stable Ba/F3 | T3065, AbmGood | Blood, lymphocyte | MERTK |

**Table S2. Intrinsic clearance**

| **Compound** | **HLM Cl_int_ (µL/min*mg)** | **MLM Cl_int_ (µL/min*mg)** | **Mouse Hepatocytes Cl_int_ (µL/min*10^6^ cells)** |
| --- | --- | --- | --- |
| Verapamil | 96.4 ± 5.5l | 230.16 ± 14.5 | 173 ± 58.7 |
| Warfarin | 4.1 ± 1.6 | 4.6 ± 0.2 | 4 ± 3.8 |
| CPL423 | 21.9 ± 0.8 | 126.5 ± 7.9 | 105.7 ± 14.6 |

**Table S3. Passive permeability in apical to basal transport**

| **Compound** | **Apparent passive permeability A to B**  **P_aap_ (10^-6^ cm/s)** | | |
| --- | --- | --- | --- |
|  | Min | Max | Avr. ± SD |
| Atenolol | 0.08 | 0.19 | 0.117 ± 0.064 |
| Propranolol | 18.41 | 29.58 | 22.23 ± 5.06 |
| CPL423 | 8.48 | 10.33 | 9.507 ± 0.942 |

**Table S4. Pharmacokinetic parameters for CPL423 in BALB/c mice**

| **Parameter** | ***p.o.* dose (10 mg/kg)**  **plasma** | ***i.v.* dose (1 mg/kg)**  **plasma** |
| --- | --- | --- |
| C_max_ (ng/ml) | 885 | 894.5 |
| t_max_ | 2 h | 8 min |
| t_1/2_ (h) | 8 | 4 |
| AUC (ng*h/ml) | 3935.6 | 616.79 |
| CL (L/h/kg) | 2.25 | 1.62 |
| *F* (%) | 67,1 | 100 |
